# Shape-Stabilized Phase Change Material via In Situ Solid–Liquid Host–Guest Composite Strategy

**DOI:** 10.3390/molecules30163376

**Published:** 2025-08-14

**Authors:** Jian Chen, Afang Zhang

**Affiliations:** International Joint Laboratory of Biomimetic and Smart Polymers, School of Materials Science and Engineering, Shanghai University, Mailbox 152, Shangda Rd. 99, Shanghai 200444, China; jchen2023@sinano.ac.cn

**Keywords:** phase change materials, shape stabilization, porous materials, nylon composites, thermal energy storage, infrared stealth

## Abstract

Solid–liquid phase change materials (PCMs) have attracted significant attention due to their high enthalpy, which enables superior energy storage density. However, it is difficult to maintain their original shapes in a molten state. Therefore, confining PCMs within porous materials is an important method, either through mixing molten polymers and PCMs or confining PCMs in pre-prepared porous materials (e.g., aerogels). The former method is straightforward and easy to execute but its stability is severely limited, and the latter is exactly the opposite. Herein, aerogel-confined functional liquid made via in situ solid–liquid host–guest composite strategy is reported. As a proof of concept, Nylon 66 and 1,6-hexanediol are selected as the solid and liquid phases, respectively. 1,6-hexanediol not only serves as a solvent to dissolve Nylon 66 but also induces sol–gel transition during the cooling process and acts as a PCM to store energy. Unlike aerogel-supported systems requiring multi-step processing, this approach integrates porous host formation and PCM encapsulation in one step. The resulting shape-stabilized PCMs (ss-PCMs) exhibit obscure leakage, high latent heat (160 J/g), mechanical robustness (compressive modulus of 3.6 MPa), and low thermal conductivity (0.081 W/(m·K)) above 75 wt% loading of 1,6-hexanediol. These ss-PCMs enable infrared stealth by delaying thermal detection and passive thermal buffering that suppress temperature fluctuations. The in situ solid–liquid host–guest composite strategy is straightforward, being achievable through a one-pot method involving heating and cooling cycles, with high raw material utilization and minimal waste generation, thus maximizing the conversion rate of raw materials into the final product. By combining the excellent liquid retention capability of aerogels with process simplicity, this methodology opens new avenues for the development of ss-PCMs.

## 1. Introduction

Phase change materials (PCMs) offer immense potential for thermal energy storage (TES) and management by leveraging reversible latent heat absorption/release during phase transitions [1,2]. However, the widespread application of solid–liquid PCMs, particularly organic types, is significantly hindered by inherent challenges due to liquid leakage in a molten state. To prevent the leakage, shape-stabilized composite PCMs (ss-CPCMs) have been developed by encapsulating PCMs within porous supports [3,4,5]. Among these, aerogels—renowned for their ultrahigh porosity (>90%), ultralow density, large specific surface area, and tunable properties—have emerged as premier supporting materials, enabling high PCM loading capacity while effectively prevent leakage via strong capillary forces and surface tension [6,7]. Carbon aerogels (graphene, biomass-derived), silica aerogels, polymer aerogels, and emerging MXene or BN aerogels have been extensively explored for this purpose [8,9,10,11,12,13,14,15,16,17,18]. Despite their advantages, the conventional fabrication of aerogel-based ss-CPCMs typically involves complex, multi-step processes in synthesis of the aerogel scaffold (e.g., sol–gel reaction, aging, supercritical drying, freeze-drying, or ambient pressure drying), followed by PCM infiltration (e.g., melt impregnation, vacuum-assisted impregnation) [19]. This route often necessitates solvent exchange and energy-intensive drying steps, which can lead to scaffold shrinkage and structural collapse, increase production cost and environmental concerns, and therefore, pose significant barriers to scalable manufacturing and practical applications.

Meanwhile, some researchers have adopted the method of melt blending phase change materials with polymers to prepare ss-CPCMs [20,21,22]. Compared to the two-step method of encapsulating PCMs in porous substrates, this method does not entail the sol–gel transition, featuring a straightforward process, but the confinement effect is often limited. Furthermore, PCMs were dissolved in solvents or dispersants before the sol–gel transition [23,24,25]. This method simplifies the preparation process and enables porous materials to effectively confine PCMs; however, this incomplete design has led to a series of problems: (1) Insufficient purity. The sol–gel transition driven by chemical reactions was chosen, which means there are many residual unreacted reactants and undesired by-products remaining in the gel. However, there was no solvent replacement step, and only a partial removal through volatilization could be achieved, causing excessive unwanted residues in the material. (2) Low volumetric specific enthalpy. Due to factors such as PCM’s solubility and sol’s stability, the concentration of PCM is usually quite low. Thus, the liquid phase is still dominated by the original solvent that turns into pores in the final step. Although the quality enthalpy change is still acceptable, the volumetric specific enthalpy will be low because of the low density. (3) Contraction or high energy consumption. As mentioned above, the liquid phase in gel is still dominated by the original solvent. If the solvent is removed through thermal evaporation, the gel framework with insufficient stiffness will collapse under the influence of capillary force, resulting in a significant shrinkage of the sample. If freeze-drying is chosen to reduce shrinkage, many solvents (such as ethanol) cannot be processed by freeze-drying; meanwhile, the use of freeze-drying means a cumbersome drying process and high energy consumption.

Herein, we present a novel and highly efficient strategy for fabricating ss-CPCMs that circumvents the above limitations. We exploit the unique behavior of Nylon (polyamide) in 1,6-hexanediol. At elevated temperatures, Nylon dissolves completely in the molten diol. Upon cooling, the Nylon crystallizes in situ, spontaneously forming a robust, interpenetrating physical network that effectively confines the 1,6-hexanediol PCM. This one-step process eliminates the need for separate aerogel synthesis, solvent exchange, and drying steps inherently involved in traditional methodologies. The resulting monolithic composite leverages the crystallized Nylon network as the supporting framework, providing exceptional shape stability to prevent leakage of the molten diol while maintaining high PCM loading density. This approach offers a significant simplification over aerogel-based confinement strategies, promising enhanced process efficiency, reduced cost, and easier scalability for high-performance thermal energy storage applications.

## 2. Results and Discussion

**Figure 1:**
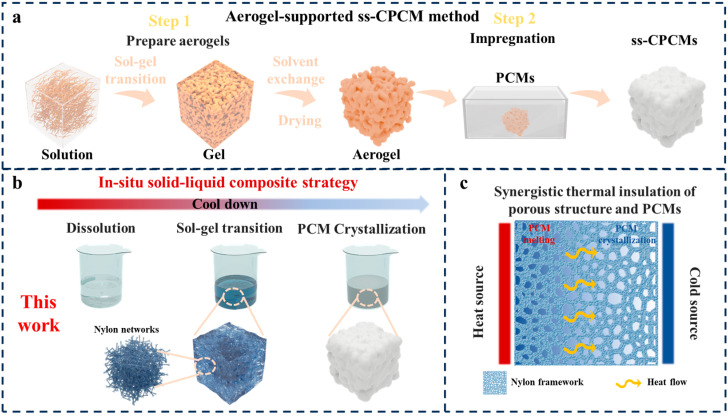
Schematic diagrams for preparing ss-CPCMs by the traditional method (**a**) and in situ solid–liquid host–guest composite strategy (**b**), as well as the synergistic thermal management of PCMs and porous structure (**c**).

Figure 1 shows conventional methodologies for synthesis of aerogel-supported ss-CPCM with our in situ strategy. Traditional routes require separated aerogel preparation (with solvent exchange/drying) and PCM infiltration, leading to extended processing, energy loss, and structural defects (Figure 1a). In contrast, our approach integrates dissolution and crystallization in one step, forming a monolithic composite where the Nylon network immobilizes the PCM while providing thermal insulation (Figure 1b,c).

### 2.1. In Situ Formation of Porous Networks

**Figure 2:**
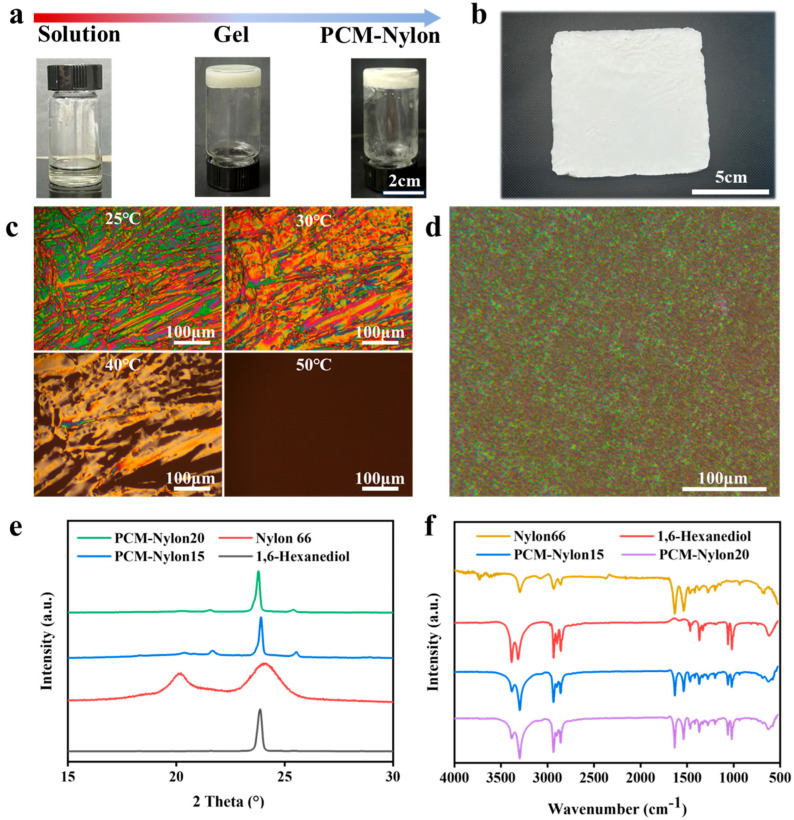
Preparation and characterization of PCM-Nylon. (**a**) Optical photographs of PCM-Nylon from high-temperature dissolution to cooling gelation. (**b**) Optical photographs of PCM-Nylon 15. (**c**) Polarizing microscope photographs of 1,6-hexanediol at different temperatures. (**d**) Polarizing microscope photographs of PCM-Nylon 15 at 25 °C. (**e**) XRD patterns of PCM-Nylon, 1,6-hexanediol, and Nylon framework. (**f**) FTIR spectra of PCM-Nylon, 1,6-hexanediol, and Nylon framework.

Figure 2a illustrates PCM-Nylon formation. Dissolution at 180 °C yielded a homogeneous solution. Cooling triggered Nylon gelation and subsequent PCM crystallization. Shape stability during phase transitions is critical for leakage prevention. A typical PCM-Nylon 15 is shown in Figure 2b.

Polarized microscopy (Figure 2c,d) clarifies crystallization dynamics: Neat 1,6-hexanediol showed large crystalline domains at 25 °C that vanished when increasing to 50 °C. Conversely, PCM-Nylon 15 exhibited no birefringence at temperatures from 25 °C to 70 °C, indicating nano/microscale PCM dispersion and suppressed crystallization due to Nylon-induced confinement.

XRD results (Figure 2e) further confirm structural interactions. Neat 1,6-hexanediol showed a sharp peak at 23.9° [26], while Nylon 66 displayed α-crystalline peaks at 20.2° and 24.1° [27]. PCM-Nylon 15 and PCM-Nylon 20 retained the PCM peak (23.9°) but exhibited shifted Nylon peaks (21.6° and 25.5°). According to Bragg’s law, a right shift in the peak position (increase in θ) means a decrease in the interplanar spacing d, that is, the unit cell contracts or distorts [28]. This result is probably caused by the insertion of small 1,6-hexanediol molecules into the lattice of Nylon 66. During high-temperature dissolution, 1,6-hexanediol molecules may be partially inserted between the Nylon 66 molecular chains, replacing the original hydrogen bond positions or occupying the inter-chain voids.

Gelation occurred when cooling. If these molecules do not completely precipitate out, they will be “frozen” in the lattice. This may force the chain segments to pack more closely, resulting in a contraction of the unit cell, a decrease in the d value, and thus a right shift in the peak position.

To further explore the morphologies, FTIR characterization was performed. For 1,6-hexanediol, -OH stretching vibration causes absorption peaks at 3385.4 cm^−1^ and 3316.0 cm^−1^ [29]. Using the C-O vibration peak at 1060.0 cm^−1^ as reference, the relative peak intensity around 3385.0 cm^−1^ for PCM-Nylon 15 and PCM-Nylon 20 decreased significantly, suggesting that hydrogen bonds and intermolecular association of 1,6-hexanediol were affected, which confirms the XRD results.

### 2.2. Pore Structure-Dictated Performance

**Figure 3:**
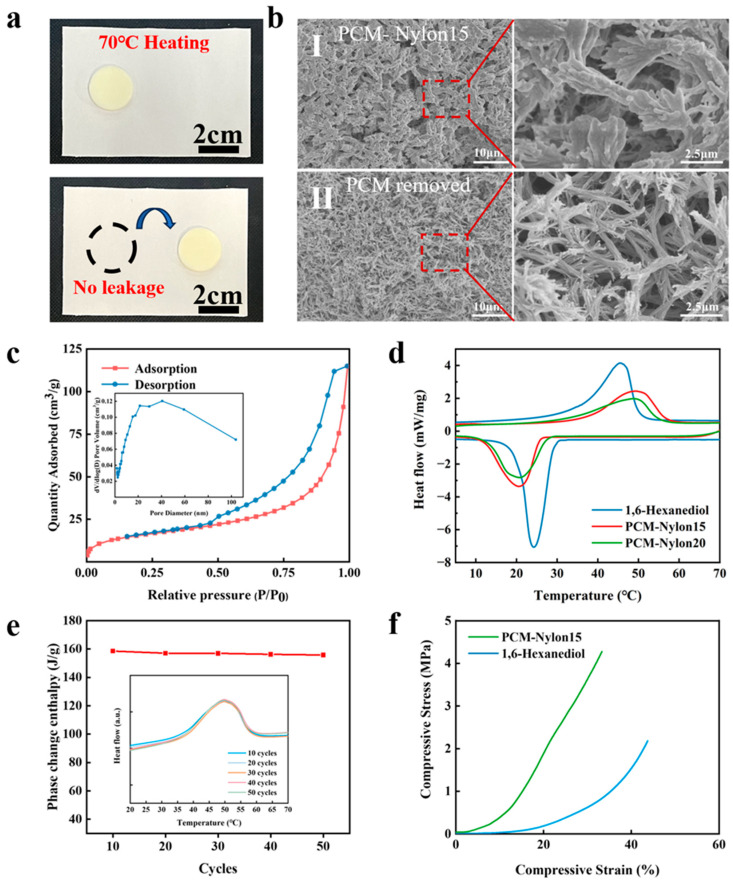
Phase change performance characterization of PCM-Nylon. Digital photographs of PCM- Nylon 15 on a 70 °C hot stage (**a**). SEM images of the Nylon framework of PCM-Nylon 15 (**I**) and PCM-Nylon 15 (**II**) (**b**). Nitrogen adsorption–desorption curves of the Nylon framework (**c**). DSC curves of 1,6-hexanediol, PCM-Nylon 15, and PCM-Nylon 20 from 5 °C to 70 °C (**d**). Phase change enthalpies and DSC curves (inset) of PCM-Nylon 15 after 10 cycles, 20 cycles, 30 cycles, 40 cycles, and 50 cycles (**e**). Mechanical compression curves of PCM-Nylon 15 and 1,6-hexanediol (**f**).

Figure 3a shows the optical photograph of PCM-Nylon 15 under a 70 °C hot stage. It maintained good dimensional stability, and no leakage of the PCM was observed. This indicates that the Nylon skeleton effectively confines 1,6-hexanediol, while provides effective support for the PCM. Figure 3b compares the morphologies of PCM-Nylon 15-f (I) with PCM-Nylon 15 (II). Inside PCM-Nylon 15-f, a sheaf-like structure of fiber aggregation was observed. Further, these sheaf-like structures overlap with each other to form a three-dimensional network structure. Inside PCM-Nylon 15, in situ solid–liquid composite phenomenon of PCM-Nylon 15-f and 1,6-hexanediol can be clearly observed. The fiber aggregation, the tip bifurcation of the sheaf, and the overlap between sheaves all form a large number of pores. These pores have extremely high capillary forces and can effectively confine 1,6-hexanediol. Results of the nitrogen adsorption–desorption curves (Figure 3c) confirm this inference. The BET specific surface area of PCM- Nylon 15-f is 55.8 m^2^/g and the average pore diameter is 12.7 nm. It has an obvious mesoporous structure.

Figure 3d compares the phase transition behaviors of 1,6-hexanediol, PCM-Nylon 15, and PCM-Nylon 20 via DSC analysis. The melting onset temperature (T_m_) of pure 1,6-hexanediol occurred at 35.5 °C, while its crystallization onset temperature (T_c_) appeared at 29.0 °C. For PCM-Nylon 15, T_m_ and T_c_ shifted to 38.3 °C and 25.9 °C, respectively. Similarly, PCM-Nylon 20 exhibited T_m_ = 36.8 °C and T_c_ = 26.4 °C. This significant hysteresis probably arises from two synergistic mechanisms namely nanoconfinement effects and enhanced intermolecular interactions. The porous Nylon framework restricts molecular mobility of 1,6-hexanediol within mesopores (12.7 nm average, Figure 3c), elevating the energy barrier for solid–liquid transition during melting and inhibiting nucleation during crystallization. In addition, hydrogen bonding between 1,6-hexanediol and Nylon chains (FTIR evidence, Figure 2f) strengthens cohesive forces, requiring additional energy input for melting while delaying crystallization.

On the other hand, introduction of Nylon has reduced the phase change enthalpy to a certain extent. The phase change enthalpies of PCM-Nylon 15 and PCM-Nylon 20 are 160.0 J/g and 148.5 J/g, respectively, which are lower than that of 1,6-hexanediol (208.5 J/g). This is because Nylon does not participate in the phase change. Therefore, the higher the proportion of Nylon, the lower the phase change enthalpy. To obtain a relatively high phase change while maintaining dimensional stability, we chose PCM-Nylon 15 as the subject for subsequent experiments. Figure 3e shows the stability of PCM-Nylon 15. After 50 cycles, the phase change enthalpy of PCM-Nylon 15 decreased slightly, from about 160 J/g to 155.8 J/g. This may be related to the volatilization of 1,6-hexanediol during the cycling process. Overall, the change is not significant, indicating good thermal stability for PCM-Nylon 15. Figure 3f compares the compression properties of PCM-Nylon 15 and 1,6-hexanediol. The compression modulus of PCM-Nylon 15 is as high as 3.6 MPa, and the compression stress reaches 3.6 MPa under a 30% compression deformation. In contrast, the compression modulus of 1,6-hexanediol is only 0.91 MPa, and the compression stress is only 0.62 MPa under a 30% compression strain. Good compression stress is of great significance for the practical application of phase change composite materials.

### 2.3. Multifunctional Applications

#### 2.3.1. Infrared Stealth

Phase change composite materials are endowed with potential applications in numerous domains. Among these applications, infrared stealth assumes a pivotal role. The manipulation of surface temperature via thermal insulation and heat flow regulation emerges as an effective strategy for realizing the infrared stealth and thermal camouflage of an object [30].

**Figure 4:**
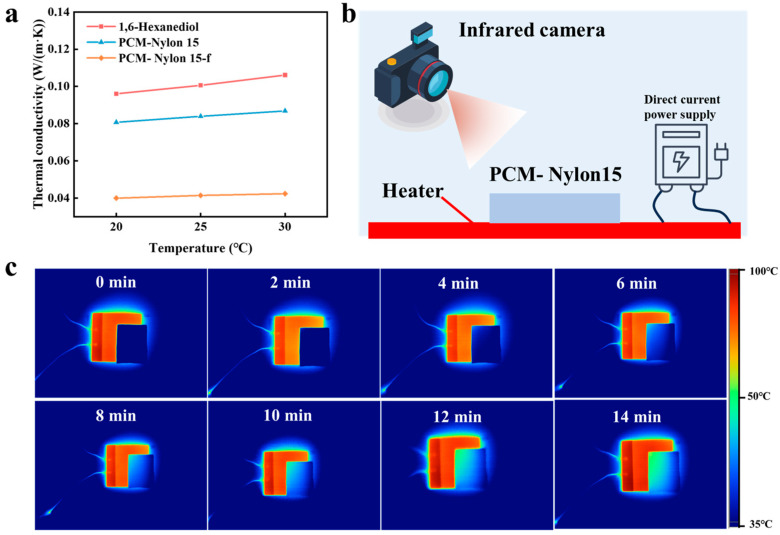
Infrared stealth performance of PCM-Nylon 15. The thermal conductivities of PCM-Nylon 15, PCM-Nylon 15-f, and 1,6-hexanediol at 20 °C, 25 °C, and 30 °C (**a**). Schematic diagram of infrared stealth experiment (**b**). The change in the infrared image of PCM-Nylon 15 placed on a 100 °C hot stage over time (**c**).

We first characterized the thermal insulation properties of 1,6-hexanediol, PCM-Nylon 15, and PCM-Nylon 15-f (Figure 4a). The thermal conductivities of 1,6-hexanediol, PCM-Nylon 15, and PCM-Nylon 15-f at 20 °C are 0.096 W/(m·K), 0.081 W/(m·K), and 0.040 W/(m·K), respectively. PCM-Nylon 15 can be regarded as a composite of 1,6-hexanediol and PCM-Nylon 15-f. Based on the geometric mean model documented in the literature [31], the thermal conductivity of the composite material can be calculated using the following formula:.

$$k_{c}={k_{a}}^{\varphi }\cdot {k_{b}}^{1-\varphi }$$

where $k_{c}$ is thermal conductivity of the composite material, $k_{a}$ is thermal conductivity of the matrix material, $k_{b}$ is thermal conductivity of the loaded material, and φ is volume fraction of the matrix material. For PCM-Nylon 15, 1,6-hexanediol occupies the majority of the phase change material volume. Thus, the thermal conductivity of PCM-Nylon 15 should lie between that of 1,6-hexanediol and PCM-Nylon 15-f, while being closer to that of 1,6-hexanediol. This prediction aligns with experimental results.

To characterize the infrared stealth performance of PCM-Nylon 15, we placed a PCM- Nylon 15 sample with a height of 4 mm on a 100 °C heater. An infrared camera was used to record the infrared imaging of PCM-Nylon 15 (Figure 4b). Within the initial six minutes, it was observable that the area covered by PCM-Nylon 15 completely merged with the background. As time elapsed, the heat on the lower surface of PCM-Nylon 15 gradually conducted to the upper surface, thereby triggering a temperature change. After eight minutes, some of the areas covered by PCM-Nylon 15 began to emerge from the background; it was not until twelve minutes later that most of the areas of PCM-Nylon 15 were revealed from the background.

#### 2.3.2. Thermal Buffering

**Figure 5:**
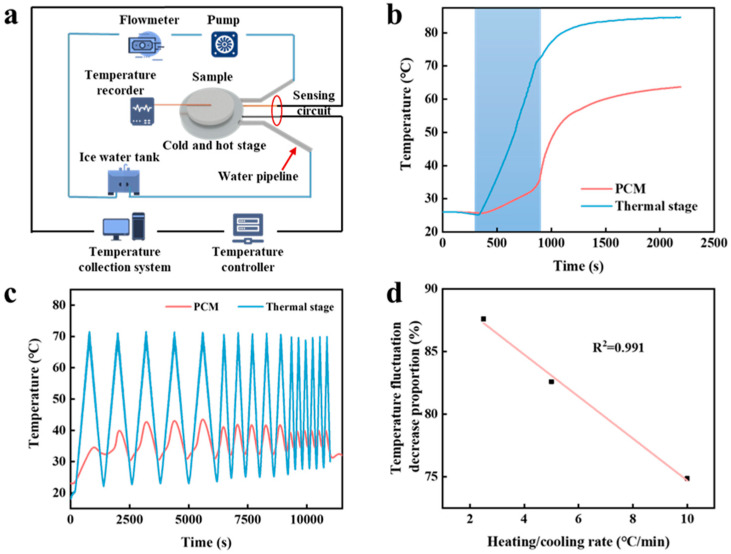
The thermal management performance of PCM-Nylon 15. Schematic diagram of the thermal management experiment (**a**). Temperature–time curve of PCM-Nylon 15 as the hot stage temperature rises (**b**). Temperature fluctuation procedure within a range of 45 °C (from 25 to 70 °C) at different rates and the corresponding temperature–time curve after applying the PCM-Nylon 15 (**c**). The temperature fluctuation decreases proportion as a function of heating/cooling rate (**d**).

Furthermore, we explored the thermal protection performance of PCM-Nylon 15. By combining a programmable temperature-controlled hot and cold stage with a temperature recorder, we recorded in real time the temperature changes on the upper and lower surfaces of PCM-Nylon 15 (Figure 5a). Figure 5b shows the temperature change in the upper surface of PCM-Nylon 15 as the hot stage temperature increased from 25 °C to 85 °C. The heating curve of the upper surface of PCM-Nylon 15 exhibited two distinct stages. In the blue-background area of the figure, the temperature of PCM-Nylon 15 increased slowly. During this stage, the 1,6-hexanediol inside PCM-Nylon 15 melted and absorbed heat. Subsequently, the slope of the temperature curve of PCM-Nylon 15 was basically consistent with that of the hot stage temperature curve. The final equilibrium temperature of the hot stage remained at around 85 °C, while the final equilibrium temperature of the upper surface of PCM-Nylon 15 was approximately 64 °C, which was attributed to the excellent thermal insulation performance of PCM-Nylon 15. Subsequently, PCM-Nylon 15 was placed in an alternating environment of 25 °C to 70 °C to investigate its thermal performance under different heating and cooling rates of 10 °C/min, 5 °C/min, and 2.5 °C/min during the cycling process. By comparing the temperature of the hot stage with that of the upper surface of PCM-Nylon 15 (Figure 5c), we found that PCM-Nylon 15 could effectively suppress temperature fluctuations. In practical applications, it could effectively protect the target from external interferences. We also found that the degree of suppression of temperature fluctuations by PCM-Nylon 15 had a linear relationship with the heating and cooling rates. The more intense the fluctuations, the more effective PCM-Nylon 15 could suppress them (Figure 5d).

### 2.4. Recyclability and Reversible Processability

**Figure 6:**
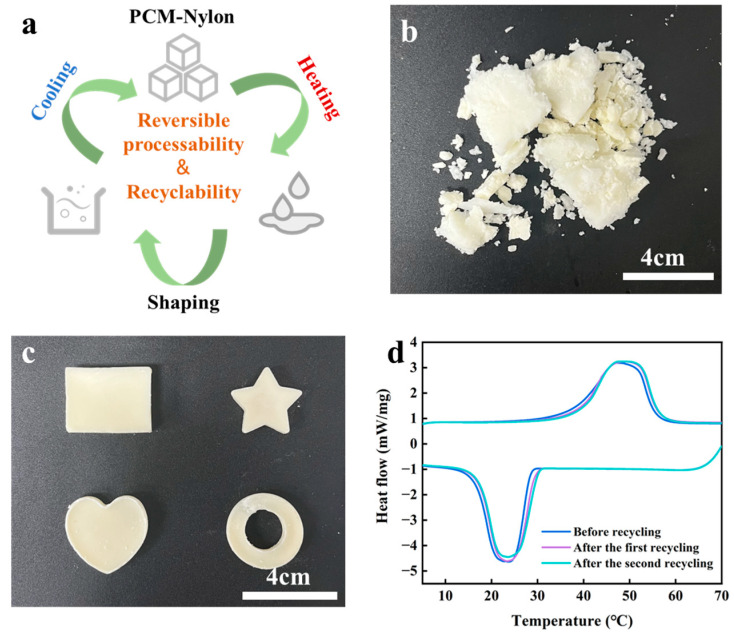
Recyclability and reversible processability of PCM-Nylon. Schematic diagram of the Recyclability and reversible processability of PCM-Nylon (**a**). Optical photographs of the PCM-Nylon 15 damaged by extrusion (**b**). Optical photographs of PCM-Nylon obtained by dissolving upon heating and reshaping upon cooling (**c**). DSC curves before recycling, after the first recycling, and after the second recycling (**d**).

PCMs are often subject to damage to the main structure under long-term use or external stress impacts, losing their original shape and dimensions, thus facing the problem of being discarded. PCM-Nylon has the characteristics of dissolving upon heating and gelling upon cooling. Therefore, it can easily achieve reprocessing plasticity and the recycling of waste PCM-Nylon, as shown in Figure 6a. Figure 6b shows PCM-Nylon 15 damaged under external stress. Through the dissolution and reshaping process described in Figure 6a, the preparation of PCM-Nylon 15 with different shapes and dimensions in Figure 6c was achieved, as well as the recycling and reuse of waste PCM-Nylon 15. The characteristics of recyclability and reversible processability make PCM-Nylon not only beneficial to environmental protection but also reduce resource consumption. Moreover, during the actual use process, it can easily meet the requirements of different shapes and dimensions. Comparison of the DSC curves before and after two recycling operations preliminarily indicates that melt recycling has no significant effect on the phase transition properties of the sample in the short term (Figure 6d). However, following numerous recycling operations over an extended period, a decline in performance may transpire. This phenomenon can be primarily ascribed to the volatilization of 1,6-hexanediol and the thermal degradation of Nylon. Notwithstanding, the service life can be extended to a certain extent. Specifically, during the recycling process, the addition of supplementary 1,6-hexanediol, coupled with the implementation of nitrogen protection during the dissolution stage, can contribute to alleviating this performance decline.

## 3. Materials and Methods

### 3.1. Materials

Nylon 66 (ERP27) was purchased from Pingdingshan Shenma Engineering PLASTICS Co., Ltd. (Pingdingshan, China). 1,6-Hexanediol (≥99%) was purchased from Shandong Yousuo Chemical Technology Co., Ltd. (Heze, China). Anhydrous ethanol (99.5%) was purchased from Aladdin Industrial Corporation (Shanghai, China). Deionized water with a resistivity of 18.2 MΩ∙cm^−1^ was obtained from a Milli-Q system (Millipore, Burlington, MA, USA). All other reagents were used without further purification.

### 3.2. Preparation of PCM-Nylon

Nylon pellets were first dried in an oven at 80 °C for 24 h. 1,6-hexanediol (2.5 mL) was mixed with Nylon 66 pellets (0.4235 g and 0.6000 g, respectively). Then, the mixtures were placed in an oil bath at 180 °C and magnetically stirred until homogeneous and transparent solutions were formed. Subsequently, they were cooled to gel at room temperature, and phase change material composites of Nylon 66 15 wt% (PCM-Nylon 15), and 20 wt% (PCM-Nylon 20) were obtained, respectively.

### 3.3. Preparation of PCM-Nylon 15 Framework (PCM-Nylon 15-f)

PCM-Nylon 15 was placed in ethanol for solvent replacement. After the replacement was completed, the sample was put in a drying kettle. Then, supercritical carbon dioxide was used for drying at 40 °C under a pressure of 10 MPa.

### 3.4. Polarizing Microscope Images

Polarized images were captured by the optical microscope (ZXPM-500, ZHXI, Shanghai, China). The polarizing microscope images at different temperatures rely on a microscopic heating and cooling stage (RTL450, HZ Instruments, Hangzhou, China) to control the temperature.

### 3.5. X-Ray Diffraction (XRD)

An X-ray Powder diffractometer (D8 ADVANCE, Bruker AXS, Karlsruhe, Germany) was employed to obtain the XRD patterns.

### 3.6. Fourier Transform Infrared (FT-IR)

FT-IR spectra were obtained via a Fourier transform infrared spectrometer (Nicolet 6700, Thermos Fisher Scientific, Waltham, MA, USA).

### 3.7. Scanning Electron Microscopy (SEM)

The microstructure was characterized via scanning electron microscopy (SEM, S4800, Hitachi High-Tech, Tokyo, Japan), which operated at an acceleration voltage of 5–10 kV, following gold sputtering on the samples’ surface.

### 3.8. Differential Scanning Calorimetry (DSC)

The melting point and enthalpy of phase transition were measured by a differential scanning calorimeter (DSC 200 F3 Maia^®^, Netzsch, Selb, Germany), with a heating and cooling rate of 10 °C/min (±0.1 K temperature accuracy, ±1% heat flow accuracy). Heat flow calibration was performed using indium (heat of fusion, 28.4 J/g). Temperature calibration was performed using indium (melting points at 156.6 °C).

### 3.9. Nitrogen Adsorption–Desorption

The ASAP 2020 instrument (Micromeritics, Norcross, GA, USA) was employed to obtain the pore size distribution by the BJH nitrogen adsorption and desorption method. As for the specific surface area of the aerogels, it was determined by the Brunauer–Emmett–Teller method, which relied on the amount of N_2_ absorbed at pressures in the range of 0.05 < P/P_0_ < 0.3.

### 3.10. Compressive Strength

Compressive curves were recorded using a universal material testing machine (3365, Instron, Norwood, MA, USA). The sample were in cylinder shape with diameter of 2.5 cm and height of 5 mm.

### 3.11. IR Images

The infrared thermal images were taken by the infrared camera (TiX580, Fluke, Everett, WA, USA) and analyzed with Smart View.

### 3.12. Thermal Conductivity

The thermal conductivity was measured using a plane table thermo-conductivity meter (HFM 436, Netzsch, Selb, Germany). The sample size is 11 cm × 11 cm × 1 cm.

### 3.13. Temperature Recording

The time–temperature curve is obtained through a multi-channel data recorder (TP900, TOPRIE, Shenzhen, China).

## 4. Conclusions

This work demonstrates that in situ solid–liquid assembly of Nylon 66 and 1,6-hexanediol enables straightforward fabrication of leakage-proof phase change composites with hierarchically porous networks. The crystallization-induced pore formation mechanism generates a robust framework (55.8 m^2^/g specific surface area) that confines PCM through synergistic capillary action and hydrogen bonding, eliminating the need for aerogel scaffolds. Optimized composites (such as PCM-Nylon 15) exhibit exceptional thermal storage capacity (160 J/g), obscure leakage at 70 °C, and mechanical strength surpassing pure PCM by 4-fold. Critically, the porous architecture imparts multifunctionality. Ultralow thermal conductivity (0.081 W·m^−1^·K^−1^) enables infrared stealth by delaying thermal detection, while passive thermal buffering suppresses temperature fluctuations by 40–60% under rapid cycling. This strategy transcends the limitations of conventional PCM supports, offering a scalable pathway toward high-performance thermal management materials.

## Data Availability

The original contributions presented in this study are included in the article. Further inquiries can be directed to the corresponding author.

## Abbreviations

The following abbreviations are used in this manuscript:

PCM	Phase change material
ss-CPCMs	Shape-stabilized composite PCMs
TES	Thermal energy storage

## References

[1] Chen X., Gao H., Tang Z., Dong W., Li A., Wang G. (2020). Optimization strategies of composite phase change materials for thermal energy storage, transfer, conversion and utilization. Energy Environ. Sci..

[2] Wang G., Tang Z., Gao Y., Liu P., Li Y., Li A., Chen X. (2023). Phase change thermal storage materials for interdisciplinary applications. Chem. Rev..

[3] Gao H., Wang J., Chen X., Wang G., Huang X., Li A., Dong W. (2018). Nanoconfinement effects on thermal properties of nanoporous shape-stabilized composite PCMs: A review. Nano Energy.

[4] Zhang S., Feng D., Shi L., Wang L., Jin Y., Tian L., Li Z., Wang G., Zhao L., Yan Y. (2021). A review of phase change heat transfer in shape-stabilized phase change materials (ss-PCMs) based on porous supports for thermal energy storage. Renew. Sustain. Energy Rev..

[5] Wu M.Q., Wu S., Cai Y.F., Wang R.Z., Li T.X. (2021). Form-stable phase change composites: Preparation, performance, and applications for thermal energy conversion, storage and management. Energy Storage Mater..

[6] Pierre A.C., Pajonk G.M. (2002). Chemistry of aerogels and their applications. Chem. Rev..

[7] Liu P., Chen X., Li Y., Cheng P., Tang Z., Lv J., Aftab W., Wang G. (2022). Aerogels meet phase change materials: Fundamentals, advances, and beyond. ACS Nano.

[8] Zhang H., Xu F., Sun L., Zou Y., Chu H., Yan E. (2017). Synthesis of three-dimensional graphene aerogel encapsulated n-octadecane for enhancing phase-change behavior and thermal conductivity. J. Mater. Chem. A.

[9] Zhao J., Luo W., Kim J.-K., Yang J. (2019). Graphene oxide aerogel beads filled with phase change material for latent heat storage and release. ACS Appl. Energy Mater..

[10] Yang J., Li X., Han S., Yang R., Min P., Yu Z.-Z. (2018). High quality graphene aerogels for thermally conductive phase change composites with excellent shape stability. J. Mater. Chem. A.

[11] Wang J., Yang M., Lu Y., Jin Z., Tan L., Gao H., Fan S., Dong W., Wang G. (2016). Surface functionalization engineering driven crystallization behavior of polyethylene glycol confined in mesoporous silica for shape-stabilized phase change materials. Nano Energy.

[12] Yu Y., Xu J., Wang G., Zhang R., Peng X. (2020). Preparation of paraffin/SiO_2_ aerogel stable-stabilized phase change composites for high-humidity environment. J. Mater. Sci..

[13] Wang H., Deng Y., Wu F., Jin H., Liu Y., Zheng J. (2021). Facile in-situ fabrication of latent heat enhanced cellulose aerogel-based form-stable composite phase change materials based on dopamine modification strategy. Sol. Energy Mater. Sol. Cells.

[14] Song M., Jiang J., Zhu J., Zheng Y., Yu Z., Ren X., Jiang F. (2021). Lightweight, strong, and form-stable cellulose nanofibrils phase change aerogel with high latent heat. Carbohydr. Polym..

[15] Yang L., Yang J., Tang L.-S., Feng C.-P., Bai L., Bao R.-Y., Liu Z.-Y., Yang M.-B., Yang W. (2020). Hierarchically porous PVA aerogel for leakage-proof phase change materials with superior energy storage capacity. Energy Fuels.

[16] Wang Y., Cui Y., Shao Z., Gao W., Fan W., Liu T., Bai H. (2020). Multifunctional polyimide aerogel textile inspired by polar bear hair for thermoregulation in extreme environments. Chem. Eng. J..

[17] Lin P., Xie J., He Y., Lu X., Li W., Fang J., Yan S., Zhang L., Sheng X., Chen Y. (2020). MXene aerogel-based phase change materials toward solar energy conversion. Sol. Energy Mater. Sol. Cells.

[18] Wang B., Li G., Xu L., Liao J., Zhang X. (2020). Nanoporous boron nitride aerogel film and its smart composite with phase change materials. ACS Nano.

[19] Kong X., Nie R., Yuan J. (2025). A review of shape stabilized aerogel-based phase change materials for preparation, classification and applications. Energy Built Environ..

[20] Molefi J.A., Luyt A.S., Krupa I. (2010). Comparison of LDPE, LLDPE and HDPE as matrices for phase change materials based on a soft Fischer–Tropsch paraffin wax. Thermochim. Acta.

[21] Krupa I., Miková G., Luyt A.S. (2007). Phase change materials based on low-density polyethylene/paraffin wax blends. Eur. Polym. J..

[22] Sarı A. (2004). Form-stable paraffin/high density polyethylene composites as solid–liquid phase change material for thermal energy storage: Preparation and thermal properties. Energy Convers. Manag..

[23] Liu L., Zou X., Wang Y., Zhou W., Shi J., Ye Y., Zhao Y., Zhang H., Yu Y., Guo J. (2021). Phase change and aerogel dual functionalized composites materials with double network structure through one-step preparation of polyacrylamide/calcium alginate/polyethylene glycol. Polymer.

[24] Zhang P., Li J., Xie R., Shen J., Song L., Chen L. (2020). One-step strategy to construct GA/PEG shape-stabilized phase change material with excellent thermophysical properties. Diam. Relat. Mater..

[25] Liu P., Gao H., Chen X., Chen D., Lv J., Han M., Cheng P., Wang G. (2020). In situ one-step construction of monolithic silica aerogel-based composite phase change materials for thermal protection. Compos. Part B Eng..

[26] Han L., Ma G., Xie S., Sun J., Jia Y., Jing Y. (2017). Thermal properties and stabilities of the eutectic mixture: 1,6-hexanediol/lauric acid as a phase change material for thermal energy storage. Appl. Therm. Eng..

[27] Murthy N.S., Curran S.A., Aharoni S.M., Minor H. (1991). Premelting crystalline relaxations and phase transitions in Nylon 6 and 6,6. Macromolecules.

[28] Kyotani M. (1979). Solution crystallization of Nylon 6. J. Polym. Sci. Part B Polym. Phys..

[29] Duan X., Wu Y., Chen Z., Yang T., Cheng Y., Yu H., Huang T. (2019). In-Situ Polymerization of High-Molecular Weight Nylon 66 Modified Clay Nanocomposites with Low Apparent Viscosity. Polymers.

[30] Wu Y., Tan S., Zhao Y., Liang L., Zhou M., Ji G. (2023). Broadband multispectral compatible absorbers for radar, infrared and visible stealth applications. Prog. Mater. Sci..

[31] Kochetov R., Andritsch T., Lafont U., Morshuis P.H.F., Picken S.J., Smit J.J. (2009). Thermal behaviour of epoxy resin filled with high thermal conductivity nanopowders. Proceedings of the 2009 IEEE Electrical Insulation Conference.

